# Pathological Fracture in Odontoid Process in Multiple Myeloma

**DOI:** 10.4274/tjh.2016.0020

**Published:** 2017-06-01

**Authors:** Emre Ali Acar, Ufuk Demirci, Yüksel Pabuşçu, Hayriye Mine Miskioğlu, İsmet Aydoğdu

**Affiliations:** 1 Celal Bayar University Faculty of Medicine, Department of Internal Medicine, Manisa, Turkey; 2 Celal Bayar University Faculty of Medicine, Department of Radiology, Manisa, Turkey; 3 Celal Bayar University Faculty of Medicine, Department of Hematology, Manisa, Turkey

**Keywords:** Myeloma, Infection, Atlas, fracture

A 72-year-old male was hospitalized with suspicion of multiple myeloma (MM) due to anemia (hemoglobin: 9.7 g/dL), renal failure (glomerular filtration rate: 8 mL/min), hypercalcemia (calcium: 11.3 mg/dL), and lytic bone lesions. He was diagnosed with MM because of increase in monoclonal lambda light chain in urine immunofixation and plasma cells (70%) in bone marrow aspiration. Fluorescence in situ hybridization and cytogenetic tests were not performed. During his chemotherapy treatment, he was operated on due to left knee septic arthritis. After the operation, he did not have any complaints for 5 weeks. After this time, he had sudden pain, restriction of movement in the neck, and temporary unconsciousness. In brain and cervical tomography there was a fracture that shifted the odontoid process and C1 vertebra, causing minimal pressure on the medulla spinalis (Figures 1,2,3).

MM usually presents with anemia (73%), bone lesions (80%), and renal failure (20%-40%). Bone involvement includes diffuse lytic lesions and osteopenia, mostly seen in the skull, vertebrae, and long bones. The frequency of lesions is associated with poor prognosis [1]. Vertebral involvement is seen in 60% of MM patients. Eighty percent of this involvement is seen between the T6 and L4 levels [2]. As seen in our case, fractures of C2 vertebrae are rare. Our patient is receiving lenalidomide because he had no response to 4 cycles of vincristine, cyclophosphamide, dexamethasone chemotherapy. In conclusion, fractures of C2 vertebrae should be considered in MM patients when restriction of neck movement and temporary unconsciousness occur.

## Figures and Tables

**Figure 1:**
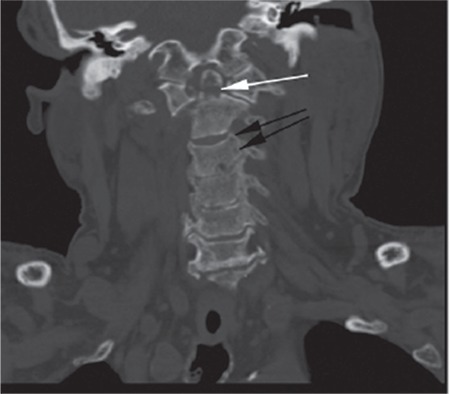
Fracture line in odontoid process (white arrow), diffuse osteolytic lesions (black arrows).

**Figure 2:**
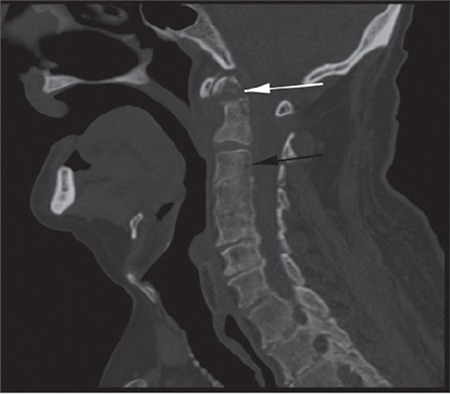
Fracture line in odontoid process (white arrow), diffuse osteolytic lesions (black arrow).

**Figure 3:**
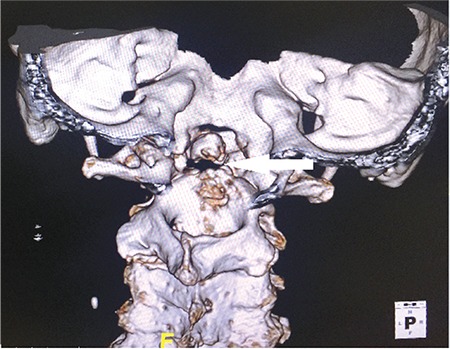
Fracture line in odontoid process in 3-dimensional cervical tomography imaging.
